# “My Attention,” Etc.

**Published:** 1896-04

**Authors:** 


"**My attention** was called to three works of art in a gallery, remarkable alike for their admirable technique and their unmitigated repulsiveness. One represented in marble the figure of the drunken god Silenus astride an ass. The only sober object in the sculpture was the ass, bestrode by the marble god, whose every fibre, muscle and feature drooped in senseless inebriety. Across the gallery was an ivory satyr, with pointed face, short horns, leering eyes, and lolling tongue, the whole expression being one of beastly sensuality. Locked in a glass case to protect it from the curious, was the head of a Bacchante, cut in the pellucid crystal of a gem bluer than God's heaven, the hair disheveled, the features distorted, the mouth open, the whole face indicating drunken frenzy.

"Give time enough and these works of art will cease to be. The marble god and the ivory satyr will disintegrate into sand and dust. But the drunken husband and father is also an artist. And he sends out into the world a hideous caricature of the living God in the person of His own child, whose life stretches away farther than our imagination can follow. It is the most serious and widespread evil of our time, the drunkenness of husbands, alike in high life and low life, and it portends the direct consequences to posterity. The woman who dares marry a libertine is doomed."—*Literary Digest.*

